# Maturation processes and structures of small secreted peptides in plants

**DOI:** 10.3389/fpls.2014.00311

**Published:** 2014-07-04

**Authors:** Ryo Tabata, Shinichiro Sawa

**Affiliations:** ^1^Graduate School of Science and Technology, Kumamoto UniversityKumamoto, Japan; ^2^Graduate School of Science, Nagoya UniversityNagoya, Japan

**Keywords:** small secreted peptide, post-translational modification, proteolytic processing, NMR structure

## Abstract

In the past decade, small secreted peptides have proven to be essential for various aspects of plant growth and development, including the maintenance of certain stem cell populations. Most small secreted peptides identified in plants to date are recognized by membrane-localized receptor kinases, the largest family of receptor proteins in the plant genome. This peptide-receptor interaction is essential for initiating intracellular signaling cascades. Small secreted peptides often undergo post-translational modifications and proteolytic processing to generate the mature peptides. Recent studies suggest that, in contrast to the situation in mammals, the proteolytic processing of plant peptides involves a number of complex steps. Furthermore, NMR-based structural analysis demonstrated that post-translational modifications induce the conformational changes needed for full activity. In this mini review, we summarize recent advances in our understanding of how small secreted peptides are modified and processed into biologically active peptides and describe the mature structures of small secreted peptides in plants.

## INTRODUCTION

Studies of small secreted peptides have flourished since the characterisation of insulin from animals in the early 1920s. The importance of small secreted peptides in cell-to-cell communication has been recognized in animals for many years. However, in plants, interest in small secreted peptides has been overshadowed by that in lipophilic non-peptide hormones, such as auxin and cytokinin (Matsubayashi and Sakagami, 2006; Betsuyaku et al., 2011; Miyawaki et al., 2013). The first plant small secreted peptide to be reported, tomato systemin (TomSys), was discovered in wounded tomato leaves (Green and Ryan, 1972; Pearce et al., 1991). This peptide activates the expression of proteinase inhibitors (Ryan, 1974), which interfere with the ability of attacking pests to digest protein (Ryan, 1990). The biochemical purification of TomSys based on its proteinase inhibitor-inducing activity led to the identification of an 18-amino acid polypeptide (Pearce et al., 1991). Over the past decade or two, the identification of several small secreted peptides has revealed that a variety of important developmental processes in plants are mediated by small secreted peptides. For example, small secreted peptides are critical players in the maintenance of stem cell populations in shoots and roots (Fletcher et al., 1999; Ito et al., 2006; Matsuzaki et al., 2010; Yamada and Sawa, 2013), in self-incompatibility (Schopfer et al., 1999; Takayama et al., 2000), and in stomatal patterning (Hara et al., 2007). In plants, small secreted peptides are mainly recognized by a membrane-associated leucine-rich repeat receptor-like kinase (LRR-RLK). Phytosulfokine (PSK), which was identified as a growth-promoting signal involved in the conditioning effect of plant cell cultures (Matsubayashi and Sakagami, 1996), was initially demonstrated to directly interact with an LRR-RLK (Matsubayashi et al., 2002). The PSK receptor, PSKR1, was purified from microsomal fractions of *Daucus carota* (carrot) cells by ligand-based affinity chromatography (Matsubayashi et al., 2002). To date, several ligand (peptide)-receptor pairs have been identified based on biochemical and genetic analyzes (Hirakawa et al., 2008; Ogawa et al., 2008; Uchida et al., 2012). This interaction between a peptide and receptor kinase is a pivotal mechanism for mediating signal input into intracellular signaling pathways.

Small secreted peptides often undergo post-translational modifications, including tyrosine sulfation, proline hydroxylation, hydroxyproline arabinosylation, and proteolytic processing, to yield the mature peptides. These processing steps are thought to affect the affinity of the peptides for their cognate receptors and their ability to activate these receptors (Ogawa et al., 2008; Okamoto et al., 2013). In this mini review, we summarize recent advances in our understanding of how small secreted peptides are modified and processed into biologically active peptides. We also describe recent NMR-based structural analyzes of plant small secreted peptides and discuss the relationship between the functional specificity and structure of small secreted peptides.

## OVERVIEW OF THE BIOSYNTHETIC PATHWAY OF SECRETED PEPTIDES

**FIGURE 1:**
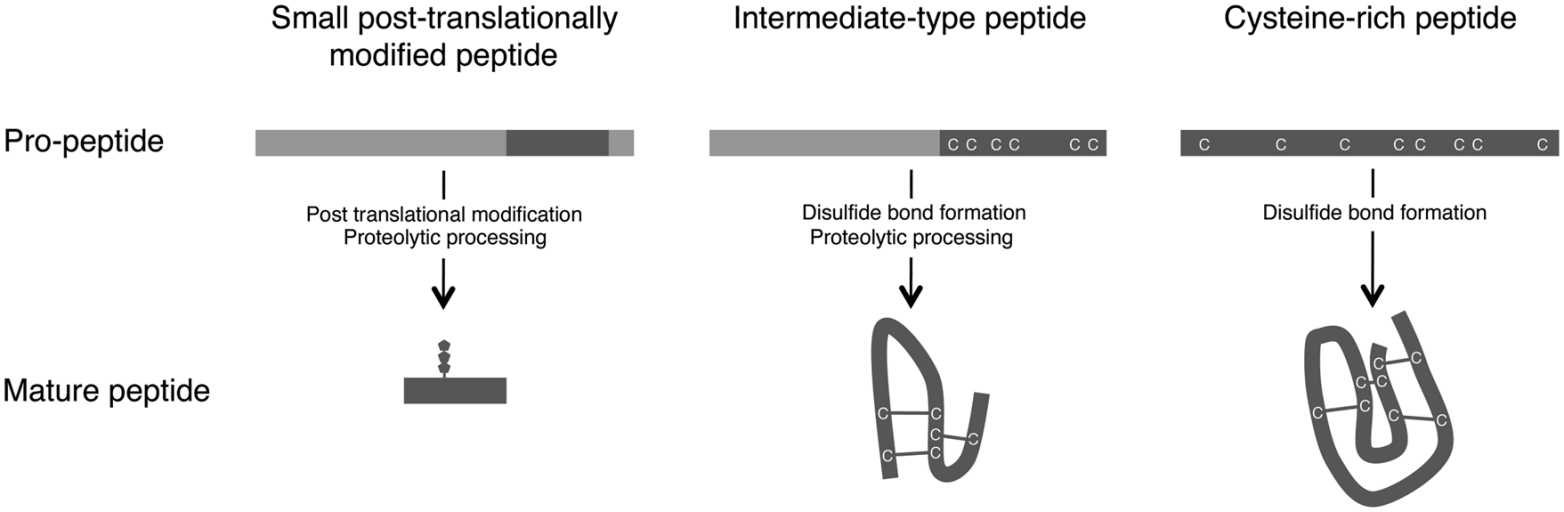
**Maturation processes of small secreted peptides.** Based on their post-translational modification and processing from pre-propeptides, small secreted peptides in plants are divided into three groups: small post-translationally modified peptides, cysteine-rich peptides, and intermediate-type peptides (Matsubayashi, 2011; Murphy et al., 2012).

Plant small secreted peptides are classified into three groups based on their biosynthetic pathway: small post-translationally modified peptides, cysteine-rich peptides, and intermediate-type peptides (Matsubayashi, 2011; Murphy et al., 2012; **Figure 1**). The genes encoding the secreted peptides are initially transcribed and then translated as pre-propeptides. This process is followed by the removal of the N-terminal signal peptide by signal peptidase. The produced propeptides are further modified by several enzymes, yielding functional mature peptides (**Figure 1**).

The first group of small secreted peptides, the small post-translationally modified peptides, consists of <20 amino acids. The propeptides corresponding to the mature peptides consist of approximately 70–120 amino acids and contain few or no cysteine residues. Several small post-translationally modified secreted peptides involved in plant growth and development have been identified, including PSK (Matsubayashi and Sakagami, 1996), PLANT PEPTIDE CONTAINING SULFATED TYROSINE 1 (PSY1; Amano et al., 2007), CLV3/EMBRYO SURROUNDING REGION-RELATED (CLE; Fletcher et al., 1999; Ito et al., 2006; Ohyama et al., 2009; Kiyohara and Sawa, 2012), C-TERMINALLY ENCODED PEPTIDE (CEP; Ohyama et al., 2008; Delay et al., 2013; Roberts et al., 2013), and ROOT GROWTH FACTOR (RGF)/GOLVEN (GLV)/CLE-LIKE (CLEL; Matsuzaki et al., 2010; Meng et al., 2012; Whitford et al., 2012).

The second group of small secreted peptides, cysteine-rich peptides, are characterized by the presence of an even number of cysteine residues (typically six or eight), which are required for the formation of disulfide bonds that maintain the mature peptide in an active conformation (**Figure 1**; Pearce et al., 2001). The cysteine-rich peptides include the S-locus cysteine-rich protein/S-locus protein 11 (SCR/SP11; Schopfer et al., 1999; Takayama et al., 2001) and LUREs (Okuda et al., 2009).

The third group, the intermediate-type peptides, is intermediate between the small post-translationally modified peptides and the cysteine-rich peptides. Although intermediate-type peptides have intramolecular disulfide bonds, these peptides are also produced via proteolytic processing (**Figure 1**). In these peptides, the cysteine residues are located within the C-terminal region of the propeptides. Stomagen, which belongs to the EPIDERMAL PATTERNING FACTOR (EPF) peptide family and is a positive regulator of stomatal density, is a representative example of this group (Hara et al., 2007; Sugano et al., 2010). Rapid alkalinization factor 1 (RALF), which is essential for cell expansion and is recognized by the FERONIA (FER) receptor, is another example of an intermediate-type peptide (Haruta et al., 2014).

## POST-TRANSLATIONAL MODIFICATIONS

Three types of post-translational modification, i.e., tyrosine sulfation, proline hydroxylation, and hydroxyproline arabinosylation, are involved in the maturation of small post-translationally modified peptides in plants.

Post-translational modification by tyrosine sulfation occurs widely in multicellular eukaryotic organisms (Kehoe and Bertozzi, 2000; Matsubayashi, 2011). Tyrosine sulfation modulates the biological activity of proteins, the proteolytic processing of bioactive peptides, and extracellular protein-protein interactions (Kehoe and Bertozzi, 2000; Matsubayashi, 2011). This type of modification is mediated by a Golgi-localised enzyme named tyrosyl protein sulfotransferase (TPST; Moore, 2003). TPST catalyzes the transfer of a sulfonate moiety from 3′-phosphoadenosine-5′-phosphosulfate (PAPS) to the hydroxyl group of a protein-bound tyrosine residue to form a tyrosine *O*-sulfate ester and 3′-phosphoadenosine-5′-phosphate (PAP; Moore, 2003). In plants, an Asp-Tyr sequence is known to be a minimum requirement for tyrosine sulfation (Matsubayashi, 2012). Plant TPST was identified by affinity purification using a PSY1-immobilized column (Komori et al., 2009). Although both animal and plant TPSTs catalyze identical sulfate transfer reactions using the same co-substrate, PAPS, they have no amino acid sequence similarity. Furthermore, plant TPST is a type I transmembrane protein with a C-terminal transmembrane domain, whereas animal TPSTs are type II transmembrane proteins with N-terminal transmembrane domains (Beisswanger et al., 1998; Ouyang et al., 1998; Komori et al., 2009). Three tyrosine-sulfated peptides, PSK, PSY1, and RGF1, have been identified in plants to date (Matsubayashi and Sakagami, 1996; Amano et al., 2007; Matsuzaki et al., 2010).

Proline hydroxylation is catalyzed by prolyl-4-hydroxylase (P4H), which is a type II membrane protein with an N-terminal transmembrane domain and is localized in both the endoplasmic reticulum (ER) and Golgi (Myllyharju, 2003). P4H is a member of a family of 2-oxoglutarate-dependent dioxygenases (Myllyharju, 2003). Thirteen genes encoding P4H have been identified in *Arabidopsis thaliana*, but no substrate consensus sequence has been established for the proline hydroxylation of secreted peptides (Matsubayashi, 2012). To date, hydroxyproline residues have been found in PSY1 (Amano et al., 2007), TDIF (Ito et al., 2006), CLV3 (Kondo et al., 2006; Ohyama et al., 2008), and RGF1 (Matsuzaki et al., 2010).

The hydroxyproline residues of several secreted peptide signals are further modified with an O-linked L-arabinose chain (tri-arabinoside) via β-1,2-bonds (Amano et al., 2007; Ohyama et al., 2009). Hydroxyproline *O*-arabinosyltransferase (HPAT) was recently identified and purified in *A*.* thaliana* (Ogawa-Ohnishi et al., 2013). HPAT is a Golgi-localised transmembrane protein that is structurally similar to members of the glycosyltransferase GT8 family and catalyzes the transfer of L-arabinose to the hydroxyl group of hydroxyproline residues (Ogawa-Ohnishi et al., 2013). Of the three *HPAT* genes present in the *A*.* thaliana* genome, *HPAT3* plays the central role in the arabinosylation of CLE peptides (Ogawa-Ohnishi et al., 2013). In addition to hydroxyproline *O*-arabinosyltransferase, the enzymes that catalyze the elongation of arabinose residues are thought to be encoded by several genes, including RRA3 (Velasquez et al., 2011) and XEG113 (Gille et al., 2009). RRA3, and XEG113, members of GT-family-77, the loss of function of which results in reduced root hair growth (Gille et al., 2009; Velasquez et al., 2011).

In cysteine-rich peptides, correct disulfide bond formation is essential for maintaining the mature peptide in an active conformation (Pearce et al., 2001). In eukaryotes, protein disulfide isomerases (PDIs) are localized to the ER and catalyze disulfide bond formation (Gruber et al., 2006). Genomic database searches have shown that there are more than 100 PDI and PDI-like (PDIL) genes in *Arabidopsis* (Houston et al., 2005). However, whether PDI and PDIL contribute to disulfide bond formation in small secreted peptides remains unclear.

## PROTEOLYTIC PROCESSING

Proteolytic processing is critical for the formation of mature functional peptides from pro-peptides of small post-translationally modified peptides and some cysteine-rich peptides. In animals, cleavage of the precursor polypeptide has been shown to occur on the C-terminal side of paired basic amino acids by subtilisin/kexin-like pro-protein and pro-hormone convertases (Rehemtulla and Kaufman, 1992). However, the peptide processing mechanisms differ between animals and plants. Specifically, mature peptides in animals are usually generated after an initial proteolytic processing step, whereas plant peptides are processed through multiple steps (Rehemtulla and Kaufman, 1992; Matsubayashi, 2012).

A recent study showed that SUPPRESSOR OF LLP1 1 (SOL1), a putative Zn^2+^ carboxypeptidase previously isolated as a suppressor of the CLE19 over-expression phenotype (Casamitjana-Martinez et al., 2003), promotes the C-terminal processing of the CLE19 proprotein to produce functional CLE19 peptide (Tamaki et al., 2013). SOL1 possesses enzymatic activity that removes the C-terminal arginine residue of CLE19 and of some other CLE proproteins *in vitro*, and the SOL1-dependent cleavage of the C-terminal arginine residue is necessary for CLE19 activity *in vivo* (Tamaki et al., 2013). Another biochemical study, using extracts from cauliflower, detected serine protease activity that cleaves the CLV3 proprotein at the 70th arginine residue, which is located in the N-terminus of the CLE domain (Ni and Clark, 2006; Ni et al., 2011). A few amino acid residues, particularly the arginine residue located at the N-terminus of the CLE domain, are thought to be crucial for CLV3 proprotein cleavage (Ni et al., 2011). Moreover, xylem fluid from *Glycine max* (soybean) and suspension culture fluid from *Medicago truncatula* (barrel medic) exhibited endoproteolytic activity that was able to produce a functional peptide from the 31-amino-acid CLE36 proprotein (Djordjevic et al., 2011). A subtilisin-like Ser protease, AtSBT1.1, is required for the processing of the PSK4 precursor (Srivastava et al., 2008). However, PSK4 could not be directly processed to the mature peptide from its precursor via AtSBT1.1 protease. These reports suggest that the proteolytic processing of plant peptides involves a number of complex steps that occur in intercellular and/or intracellular compartments (Tamaki et al., 2013).

STOMATAL DENSITY AND DISTRIBUTION (SDD1) is a subtilisin-like extracellular protease that is involved in the proteolytic processing of cysteine-rich peptides and is thought to play a role in the processing of EPF1 (Berger and Altmann, 2000). Genetic experiments showed that *epf1 sdd1* double mutant exhibited more severe abnormalities in stomatal density than either *epf1* or *sdd1* single mutants (Hunt and Gray, 2009). Thus, an additional enzyme is thought to contribute to the processing of EPF peptides (Hunt and Gray, 2009).

## STRUCTURES OF SECRETED PEPTIDES

**FIGURE 2:**
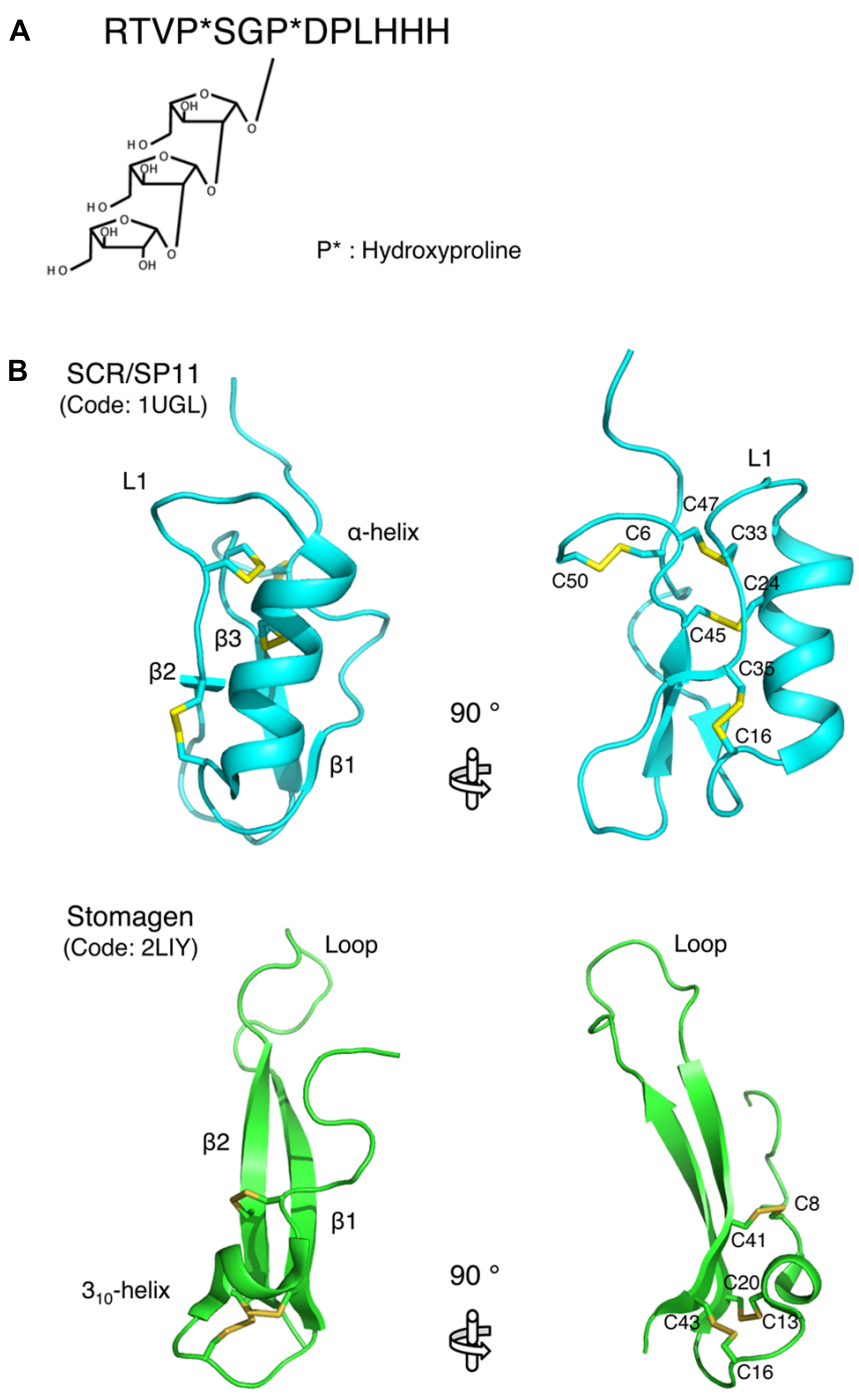
**Structures of small secreted peptides. (A)** The mature form of the CLAVATA3 peptide. Hydroxylated proline residues are indicated by asterisks. **(B)** Ribbon models of SCR/SP11 and stomagen presented from two perspectives. The disulfide bonds are depicted as ball-and-stick models with residue name. These models were generated using MOLMOL (Koradi et al., 1996).

Recent structural studies revealed the molecular basis of the biological activities of plant small secreted peptides. The active form of the CLV3 peptide, which belongs to the small post-translationally modified group of small secreted peptides, has been characterized by NMR-based structural analysis (Shinohara and Matsubayashi, 2013). Arabinosylation of the hydroxyproline residues is an essential modification for the activity of the CLV3 peptide (Ohyama et al., 2009; Shinohara and Matsubayashi, 2013; **Figure 2A**). A comparison of the structures of the non-arabinosylated CLV3 peptide and a β-1,2-linked tri-arabinosylated CLV3 peptide [(Ara_3_)CLV3] revealed that the linear arabinose chain adopts a helical conformation that turns toward the C-terminal side of the peptide (Shinohara and Matsubayashi, 2013). Thus, this hydroxyproline-bound tri-arabinoside induces a conformational alteration in the peptide backbone, turning it toward the C-terminus via steric repulsion, and contributes to the full biological activity of this peptide. The biological activity of the CLV3 peptide is progressively increased with increasing arabinose chain length (Shinohara and Matsubayashi, 2013). The structure of another small post-translationally modified peptide, *M. truncatula* CEP1 (MtCEP1), was also analyzed by NMR (Bobay et al., 2013). The solution structure shows that MtCEP1 contains a β-turn-like conformation (Bobay et al., 2013), although the correlation between the structure and biological function of this peptide is unclear. Further research should analyze the contribution of each amino acid residue to the biological activity of the peptide.

The structure of SCR/SP11, which belongs to the cysteine-rich peptide group of small peptides, has been determined. SCR/SP11 folds into an α/β sandwich motif that resembles that of plant defensins (Mishima et al., 2003; **Figure 2B**). The L1 loop connects the helix and strand β2, and this loop contains a high degree of variation depending on the insertion and deletion mutations in the encoding SCR/SP11 allele (Mishima et al., 2003; **Figure 2B**). Structure-based sequence alignment and homology modeling of SCR/SP11 variants encoded by different alleles suggest that the loop region could serve as a specific binding site for receptors (Mishima et al., 2003).

The structure of stomagen, an intermediate-type small secreted peptide, has also been determined. Stomagen, which is a member of the EPF family of peptides and is composed of a loop and a scaffold containing three disulfide bonds, contains two anti-parallel β-strands connected by a 14-residue loop (Ohki et al., 2011; **Figure 2B**). The loop spans a largely divergent region in the amino acid sequence alignment of EPF family members, and is thus referred to as the hypervariable region (Ohki et al., 2011). A loop region swapping analysis of stomagen and EPF2 demonstrated that the loop confers functional specificity on EPF peptides (Ohki et al., 2011). Similar to cysteine-rich peptides, the loop region contains charged residues that commonly occur at protein–protein interfaces (**Figure 2B**; Jones and Thornton, 1996). These findings support the hypothesis that these loop regions are crucial for the efficient and specific binding of the peptides to their receptors and thus for the biological activities of these peptides. These structural approaches to elucidating the peptide structures that contribute to activity could be adapted for the analysis of other secreted peptides.

## CONCLUSION

Small secreted peptides have critical roles in cell-to-cell communication during plant growth and development. Recent studies of secreted peptides revealed that post-translational modifications and proteolytic processing are essential for the biological activity and functional specificity of these peptides. However, our understanding of these maturation steps is incomplete. The identification of enzymes, including peptidases, involved in post-translational modification and proteolytic processing will unravel the maturation steps of plant secreted peptides. Biochemical approaches and rapid genetic screens for suppressors/enhancers, using next-generation sequencing technology may be effective strategies for identifying such proteins (Casamitjana-Martinez et al., 2003; Komori et al., 2009; Tabata et al., 2013), as was successfully demonstrated for hydroxyproline *O*-arabinosyltransferase (Ogawa-Ohnishi et al., 2013) and carbosypeptidase (Tamaki et al., 2013). Furthermore, precise spatiotemporal expression analysis of secreted peptides and the identification of the cognate receptor(s) for each peptide will provide novel insight into the molecular functions of plant small secreted peptides.

## Conflict of Interest Statement

The authors declare that the research was conducted in the absence of any commercial or financial relationships that could be construed as a potential conflict of interest.

## References

[B1] Amano Y. Tsubouchi H. Shinohara H. Ogawa M. Matsubayashi Y. (2007). Tyrosine-sulfated glycopeptide involved in cellular proliferation and expansion in *Arabidopsis*. *Proc. Natl. Acad. Sci. U.S.A.* 104 18333–18338 10.1073/pnas.0706403104 17989228 PMC2084343

[B2] Beisswanger R. Corbeil D. Vannier C. Thiele C. Dohrmann U. Kellner R. (1998). Existence of distinct tyrosylprotein sulfotransferase genes: molecular characterization of tyrosylprotein sulfotransferase-2. *Proc. Natl. Acad. Sci. U.S.A.* 95 11134–11139 10.1073/pnas.95.19.11134 9736702 PMC21608

[B3] Berger D. Altmann T. (2000). A subtilisin-like serine protease involvedin the regulation of stomatal density and distribution in *Arabidopsis thaliana*. *Genes Dev.* 14 1119–1131 10.1101/gad.14.9.1119 10809670 PMC316574

[B4] Betsuyaku S. Sawa S. Yamada M. (2011). The function of the CLE peptidein plant development and plant-microbe interactions. *Arabidopsis Book* 9:e0149 10.1199/tab.0149 PMC3268505 22303273

[B5] Bobay B. G. Digennaro P. Scholl E. Imin N. Djordjevic M. A. McK Bird D. (2013). Solution NMR studies of the plant peptide hormone CEP inform function. *FEBS Lett.* 587 3979–3985 10.1016/j.febslet.2013.10.033 24211833

[B6] Casamitjana-Martinez E. Hofhuis H. F. Xu J. Liu C. M. Heidstra R. Scheres B. (2003). Root-specific CLE19 overexpression and the sol1/2suppressors implicate a CLV-like pathway in the control of *Arabidopsis* root meristem maintenance. *Curr. Biol.* 13 1435–1441 10.1016/S0960-9822(03)00533-5 12932329

[B7] Delay C. Imin N. Djordjevic M. A. (2013). CEP genes regulate root andshoot development in response to environmental cues and are specific toseed plants. *J. Exp. Bot.* 64 5383–5394 10.1093/jxb/ert332 24179096

[B8] Djordjevic M. A. Oakes M. Wong C. E. Singh M. Bhalla P. Kusumawati L. (2011). Border sequences of *Medicago truncatula* CLE36are specifically cleaved by endoproteases common to the extracellularfluids of *Medicago* and soybean. *J. Exp. Bot.* 62 4649–4659 10.1093/jxb/err185 21633083 PMC3170558

[B9] Fletcher J. C. Brand U. Running M. P. Simon R. Meyerowitz E. M. (1999). Signaling of cell fate decisions by CLAVATA3 in *Arabidopsis* shoot meristems. *Science* 283 1911–1914 10.1126/science.283.5409.1911 10082464

[B10] Gille S. Hansel U. Ziemann M. Pauly M. (2009). Identification ofplant cell wall mutants by means of a forward chemical genetic approach using hydrolases. *Proc. Natl. Acad. Sci. U.S.A.* 106 14699–14704 10.1073/pnas.0905434106 19667208 PMC2731844

[B11] Green T. R. Ryan C. A. (1972). Wound-induced proteinase inhibitor in plant leaves: a possible defense mechanism against insects. *Science* 175 776–777 10.1126/science.175.4023.776 17836138

[B12] Gruber C. W. Cemazar M. Heras B. Martin J. L. Craik D. J. (2006). Protein disulfide isomerase: the structure of oxidative folding. *Trends Biochem. Sci.* 31 455–464 10.1016/j.tibs.2006.06.001 16815710

[B13] Hara K. Kajita R. Torii K. U. Bergmann D. C. Kakimoto T. (2007). The secretory peptide gene EPF1 enforces the stomatal one-cell-spacing rule. *Genes Dev.* 21 1720–1725 10.1101/gad.1550707 17639078 PMC1920166

[B14] Haruta M. Sabat G. Stecker K. Minkoff B. B. Sussman M. R. (2014). A peptide hormone and its receptor protein kinase regulate plant cell expansion. *Science* 343 408–411 10.1126/science.1244454 24458638 PMC4672726

[B15] Hirakawa Y. Shinohara H. Kondo Y. Inoue A. Nakanomyo I. Ogawa M. (2008). Non-cell-autonomous control of vascular stem cell fate by a CLE peptide/receptor system. *Proc. Natl. Acad. Sci. U.S.A.* 105 15208–15213 10.1073/pnas.0808444105 18812507 PMC2567516

[B16] Houston N. L. Fan C. Xiang J. Q. Schulze J. M. Jung R. Boston R. S. (2005). Phylogenetic analyses identify 10 classes of the protein disulfide isomerase family in plants, including single-domain protein disulfide isomerase-related proteins. *Plant Physiol.* 137 762–778 10.1104/pp.104.056507 15684019 PMC1065376

[B17] Hunt L. Gray J. E. (2009). The signalling peptide EPF2 controls asymmetric cell divisions during stomatal development. *Curr. Biol.* 19 864–869 10.1016/j.cub.2009.03.069 19398336

[B18] Ito Y. Nakanomyo I. Motose H. Iwamoto K. Sawa S. Dohmae N. (2006). Dodeca-CLE peptides as suppressors of plant stem cell differentiation. *Science* 313 842–845 10.1126/science.1128436 16902140

[B19] Jones S. Thornton J. M. (1996). Principles of protein-protein interactions. *Proc. Natl. Acad. Sci. U.S.A.* 93 13–20 10.1073/pnas.93.1.13 8552589 PMC40170

[B20] Kehoe J. W. Bertozzi C. R. (2000). Tyrosine sulfation: a modulator of extracellular protein-protein interactions. *Chem. Biol.* 7 R57–R61 10.1016/S1074-5521(00)00093-4 10712936

[B21] Kiyohara S. Sawa S. (2012). CLE signaling systems during plant development and nematode infection. *Plant Cell Physiol.* 53 1989–1999 10.1093/pcp/pcs136 23045524

[B22] Komori R. Amano Y. Ogawa-Ohnishi M. Matsubayashi Y. (2009). Identification of tyrosylprotein sulfotransferase in *Arabidopsis*. *Proc. Natl. Acad. Sci. U.S.A.* 106 15067–15072 10.1073/pnas.0902801106 19666544 PMC2736448

[B23] Kondo T. Sawa S. Kinoshita A. Mizuno S. Kakimoto T. Fukuda H. (2006). A plant peptide encoded by CLV3 identified by in situ MALDI-TOF MS analysis. *Science* 313 845–848 10.1126/science.1128439 16902141

[B24] Koradi R. Billeter M. Wuthrich K. (1996). MOLMOL: a program for display and analysis of macromolecular structures. *J. Mol. Graph.* 14 51–55 10.1016/0263-7855(96)00009-4 8744573

[B25] Matsubayashi Y. (2011). Post-translational modifications in secreted peptide hormones in plants. *Plant Cell Physiol.* 52 5–13 10.1093/pcp/pcq169 21071428 PMC3023852

[B26] Matsubayashi Y. (2012). MBSJ MCC Young Scientist Award 2010. Recent progress in research on small post-translationally modified peptide signals in plants. *Genes Cells* 17 1–10 10.1111/j.1365-2443.2011.01569.x 22212512

[B27] Matsubayashi Y. Sakagami Y. (1996). Phytosulfokine, sulfated peptides that induce the proliferation of single mesophyll cells of Asparagus officinalis L. *Proc. Natl. Acad. Sci. U.S.A.* 93 7623–7627 8755525 10.1073/pnas.93.15.7623 PMC38796

[B28] Matsubayashi Y. Sakagami Y. (2006). Peptide hormones in plants. *Annu. Rev. Plant Biol.* 57 649–674 10.1146/annurev.arplant.56.032604.144204 16669777

[B29] Matsubayashi Y. Ogawa M. Morita A. Sakagami Y. (2002). An LRR receptor kinase involved in perception of a peptide plant hormone, phytosulfokine. *Science* 296 1470–1472 10.1126/science.1069607 12029134

[B30] Matsuzaki Y. Ogawa-Ohnishi M. Mori A. Matsubayashi Y. (2010). Secreted peptide signals required for maintenance of root stem cell niche in *Arabidopsis*. *Science* 329 1065–1067 10.1126/science.1191132 20798316

[B31] Meng L. Buchanan B. B. Feldman L. J. Luan S. (2012). CLE-like (CLEL) peptides control the pattern of root growth and lateral root development in *Arabidopsis*. *Proc. Natl. Acad. Sci. U.S.A.* 31 1760–1765 10.1073/pnas.1119864109 22307643 PMC3277184

[B32] Mishima M. Takayama S. Sasaki K. Jee J. G. Kojima C. Isogai A. (2003). Structure of the male determinant factor for *Brassica* self-incompatibility. *J. Biol. Chem.* 278 36389–36395 10.1074/jbc.M305305200 12835321

[B33] Miyawaki K. Tabata R. Sawa S. (2013). Evolutionarily conserved CLE peptide signaling in plant development, symbiosis, and parasitism. *Curr. Opin. Plant Biol.* 16 598–606 10.1016/j.pbi.2013.08.008 24035739

[B34] Moore K. L. (2003). The biology and enzymology of protein tyrosine O-sulfation. *J. Biol. Chem.* 278 24243–24246 10.1074/jbc.R300008200 12730193

[B35] Murphy E. Smith S. De Smet I. (2012). Small signaling peptides in *Arabidopsis* development: how cells communicate over a short distance. *Plant Cell* 24 3198–3217 10.1105/tpc.112.099010 22932676 PMC3462626

[B36] Myllyharju J. (2003). Prolyl 4-hydroxylases, the key enzymes of collagen biosynthesis. *Matrix Biol.* 22 15–24 10.1016/S0945-053X(03)00006-4 12714038

[B37] Ni J. Clark S. E. (2006). Evidence for functional conservation, sufficiency, and proteolytic processing of the CLAVATA3 CLE domain. *Plant Physiol.* 140 726–733 10.1104/pp.105.072678 16407446 PMC1361338

[B38] Ni J. Guo Y. Jin H. Hartsell J. Clark S. E. (2011). Characterization of a CLE processing activity. *Plant Mol. Biol.* 75 67–75 10.1007/s11103-010-9708-2 21052783

[B39] Ogawa M. Shinohara H. Sakagami Y. Matsubayashi Y. (2008). Arabidopsis CLV3 peptide directly binds CLV1 ectodomain. *Science* 319 294 10.1126/science.1150083 18202283

[B40] Ogawa-Ohnishi M. Matsushita W. Matsubayashi Y. (2013). Identification of three hydroxyproline O-arabinosyltransferases in *Arabidopsis thaliana*. *Nat. Chem. Biol.* 9 726–730 10.1038/nchembio.1351 24036508

[B41] Ohki S. Takeuchi M. Mori M. (2011). The NMR structure of stomagen reveals the basis of stomatal density regulation by plant peptide hormones. *Nat. Commun.* 2 512 10.1038/ncomms1520 22027592

[B42] Ohyama K. Ogawa M. Matsubayashi Y. (2008). Identification of a biologically active, small, secreted peptide in *Arabidopsis* by in silico gene screening, followed by LC-MS-based structure analysis. *Plant J.* 55 152–160 10.1111/j.1365-313X.2008.03464.x 18315543

[B43] Ohyama K. Shinohara H. Ogawa-Ohnishi M. Matsubayashi Y. (2009). A glycopeptide regulating stem cell fate in *Arabidopsis thaliana*. *Nat. Chem. Biol.* 5 578–580 10.1038/nchembio.182 19525968

[B44] Okamoto S. Shinohara H. Mori T. Matsubayashi Y. Kawaguchi M. (2013). Root-derived CLE glycopeptides control nodulation by direct binding to HAR1 receptor kinase. *Nat. Commun.* 4 2191 10.1038/ncomms3191 23934307

[B45] Okuda S. Tsutsui H. Shiina K. Sprunck S. Takeuchi H. Yui R. (2009). Defensin-like polypeptide LUREs are pollen tube attractants secreted from synergid cells. *Nature* 458 357–361 10.1038/nature07882 19295610

[B46] Ouyang Y. Lane W. S. Moore K. L. (1998). Tyrosylprotein sulfotransferase: purification and molecular cloning of an enzyme that catalyzes tyrosine O-sulfation, a common posttranslational modification of eukaryotic proteins. *Proc. Natl. Acad. Sci. U.S.A.* 95 2896–2901 10.1073/pnas.95.6.2896 9501187 PMC19666

[B47] Pearce G. Moura D. Stratmann J. Ryan C. A. (2001). Production of mutlitple plant hormones from a single polyprotein precursor. *Nature* 411 817–820 10.1038/35081107 11459063

[B48] Pearce G. Strydom D. Johnson S. Ryan C. A. (1991). A polypeptide from tomato leaves activates the expression of proteinase inhibitor genes. *Science* 253 895–897 10.1126/science.253.5022.895 17751827

[B49] Rehemtulla A. Kaufman R. J. (1992). Protein processing within the secretory pathway. *Curr. Opin. Biotechnol.* 3 560–565 10.1016/0958-1669(92)90086-X 1368940

[B50] Roberts I. Smith S. De Rybel B. Van Den Broeke J. Smet W. De Cokere S. (2013). The CEP family in land plants: evolutionary analyses, expression studies, and role in *Arabidopsis* shoot development. *J. Exp. Bot.* 64 5371–5381 10.1093/jxb/ert331 24179095

[B51] Ryan C. A. (1974). Assay and biochemical properties of the proteinase inhibitor inducing factor, a wound hormone. *Plant Physiol.* 54 328–332 10.1104/pp.54.3.328 16658883 PMC367406

[B52] Ryan C. A. (1990). Proteinase inhibitors in plants: genes for improving defenses against insects and pathogens. *Annu. Rev. Phytopathol.* 28 425–449 10.1146/annurev.py.28.090190.002233

[B53] Schopfer C. R. Nasrallah M. E. Nasrallah J. B. (1999). The male determinant of self-incompatibility in *Brassica*. *Science* 286 1697–1700 10.1126/science.286.5445.1697 10576728

[B54] Shinohara H. Matsubayashi Y. (2013). Chemical synthesis of *Arabidopsis* CLV3 glycopeptide reveals the impact of hydroxyproline arabinosylation on peptide conformation and activity. *Plant Cell Physiol.* 54 369–374 10.1093/pcp/pcs174 23256149 PMC3589827

[B55] Srivastava R. Liu J. X. Howell S. H. (2008). Proteolytic processing of a precursor protein fo a growth-promoting peptide by a subtilisin serine protease in *Arabidopsis*. *Plant J.* 56 219–227 10.1111/j.1365-313X.2008.03598.x 18643977 PMC2667306

[B56] Sugano S. S. Shimada T. Imai Y. Okawa K. Tamai A. Mori M. (2010). Stomagen positively regulates stomatal density in *Arabidopsis*. *Nature* 463 241–244 10.1038/nature08682 20010603

[B57] Tabata R. kamiya T. Shigenobu S. Yamaguchi K. Yamada M. Hasebe M. (2013). Identification of an EMS-induced causal mutation in a gene required for boron-mediated root development by low-coverage genome re-sequencing in *Arabidopsis*. *Plant Signal. Behav.* 8:e22534 10.4161/psb.22534 PMC3745560 23104114

[B58] Takayama S. Shiba H. Iwano M. Shimosato H. Che F. S. Kai N. (2000). The pollen determinant of self-incompatibility in *Brassica campestris*. *Proc. Natl. Acad. Sci. U.S.A.* 97 1920–1925 10.1073/pnas.040556397 10677556 PMC26537

[B59] Takayama S. Shimosato H. Shiba H. Funato M. Che F. S. Watanabe M. (2001). Direct ligand-receptor complex interaction controls *Brassica* self-incompatibility. *Nature* 413 534–538 10.1038/35097104 11586363

[B60] Tamaki T. Betsuyaku S. Fujiwara M. Fukao Y. Fukuda H. Sawa S. (2013). SUPPRESSOR OF LLP1 1-mediated C-terminal processing is critical for CLE19 peptide activity. *Plant J.* 76 970–981 10.1111/tpj.12349 24118638

[B61] Uchida N. Lee J. S. Horst R. J. Lai H. H. Kajita R. Kakimoto T. (2012). Regulation of inflorescence architecture by intertissue layer ligand-receptor communication between endodermis and phloem. *Proc. Natl. Acad. Sci. U.S.A.* 109 6337–6342 10.1073/pnas.1117537109 22474391 PMC3341066

[B62] Velasquez S. M. Ricardi M. M. Dorosz J. G. Fernandez P. V. Nadra A. D. Pol-Fachin L. (2011). O-glycosylated cell wall proteins are essential in root hair growth. *Science* 332 1401–1403 10.1126/science.1206657 21680836

[B63] Whitford R. Fernandez A. Tejos R. Perez A. C. Kleine-Vehn J. Vanneste S. (2012). GOLVEN secretory peptides regulate auxin carrier turnover during plant gravitropic response. *Dev. Cell* 22 678–685 10.1016/j.devel.2012.02.002 22421050

[B64] Yamada M. Sawa S. (2013). The roles of peptide hormones duringplant root development. *Curr. Opin. Plant. Biol.* 16 56–61 10.1016/j.pbi.2012.11.004 23219865

